# Pachychoroid neovasculopathy can mimic wet type age-related macular degeneration

**DOI:** 10.1186/s40942-022-00429-6

**Published:** 2022-10-23

**Authors:** Mohsen Farvardin, Abdulrahim Amini, Younes Azizpourfard, Masoud Yasemi, Zahra Mahdizad, Mohammadkarim Johari

**Affiliations:** 1 Poostchi Ophthalmology Research Center, Department of Ophthalmology, School of Medicine, Shiraz University of Medical Sciences, Zand Street, Shiraz, Iran; 2 Department of ophthalmology, school of medicine, Hormozgan University of medical sciences, Bandar Abbas, Iran; 3 Eye Research Center, Farabi Eye Hospital, Tehran University of medical science, Tehran, Iran

**Keywords:** Pachychoroid neovasculopathy, Macular degeneration, Choroidal neovascularization, Choroidal thickness

## Abstract

**Purpose:**

to determine the percentage of patients with pachychoroid neovasculopathy (PNV) among patients who have been misdiagnosed and treated with wet age-related macular degeneration (AMD).

**Methods:**

In this retrospective cross-sectional study, patients over 55 years old, who were diagnosed with wet AMD, were re-evaluated. All patients were recalled for examination and imaging. Patients with PNV were differentiated form wet AMD based on inclusion and exclusion criteria.

**Results:**

Overall, 120 patients (137 eyes) were recorded with wet AMD in the clinic. Finally, after complete re-evaluation, 94 (106 eyes) and 26 patients (31 eyes) were assigned to the AMD and the PNV group, respectively. Thus, a total of 20% of patients with primary mistake diagnosis of wet AMD, actually had PNV. The mean sub field choroidal thickness (SFCT) in the AMD and PNV groups was 173.8 ± 69 μm and 342 ± 27 μm, respectively. Drusen and pachydrusen were found in 69.9% and 24% of the cases with AMD and PNV, respectively (P = 0.001). The average number of intravitreal injections of anti-VEGF (vascular endothelial growth factor) required in the AMD and PNV groups was about 5 and 3, respectively, which was statistically significant (P-value 0.02).

**Conclusion:**

This study revealed that about a one-fifth of wet AMD patients are actually pachychoroid neovasculopathy. These patients were younger and had thicker SFCT, and developed less subretinal scarring. Thus, the disorder must be considered as an important differential diagnosis of AMD-CNV.

## Introduction

Age-related macular degeneration (AMD) is one of the leading causes of irreversible vision loss in developed countries, characterized by complete degeneration of retinal pigment epithelium (RPE) in early stages and retinal photoreceptors by neovascularization or geographic atrophy in later stages [1]. Choroidal tissue’s healthy structure and function for appropriate retina functioning are crucially important [1, 2]. Many studies proved the essential role of the choroidal vein network in the pathophysiology of some retinal diseases. It included central serous chorioretinopathy (CSCR), AMD, Vogt–Koyanagi–Harada disease (VKH), and pathological myopia [3, 4, 6].

Warrow et al. identified pachychoroid pigment epitheliopathy as a disorder related to pachychoroid diseases and suggested that genetic predisposition may play a role in the disease. Increased sub-field choroidal thickness (SFCT), dilated Haller layer veins, and decreased thickness of Sattler and choroicapillary layers are common characteristics of pachychoroid diseases [5].

Pang et al. described Pachychoroid neovasculopathy (PNV) as a spectrum of diseases associated with choroidal thickening, including pachychoroid pigment epitheliopathy, central serous chorioretinopathy, and polypoidal choroidal vasculopathy and it should be considered a possible diagnosis in eyes with features of Type 1 macular neovascularization.[8] Balaratnainga et al. introduced the definition of pachychoroid phenotype as having one or more of the following characteristics: increased SFCT, pathologically dilated outer choroidal vessels (pachyvessel), choriocapillaris and Sattler’s vessels attenuation accompany with Haller pachyvessel augmentation, increased choroidal vascular hyperpermeability, and decreased fundus tessellation in choroidal thickening area. [9]. Prolonged pressure on the Bruch’s membrane can lead to pachychoroid neovascularization in the Haller layer [11]. Subsequently, the first type of CNV may occur following CSCR and pachychoroid pigment epitheliopathy (PPE). This vascular network gradually expands and forms a polyp [11, 12]. One of the important differential diagnoses of PNV is AMD and other conditions that cause the first type of CNV, although clinical diagnosis is sometimes difficult [12]. PNV is often mistaken for AMD-CNV because of its phenotypic indistinguishability. [10, 12, 13]. However, thanks to the expanded use of multimodal imaging modalities and imaging methods, including indocyanine green (ICG), enhanced depth imaging optical coherence tomography (EDI-OCT), and OCT angiography help determine the SFCT and thus diagnosing related pathologies [11–13]. The present study aims to determine the percentage of patients with pachychoroid neovasculopathy (PNV) among patients who have been misdiagnosed and treated with AMD-CNV. The present study also introduces appropriate diagnostic criteria to differentiate between these two diseases.

## Methods

This retrospective cross-sectional study protocol adhered to the principles outlined in the Declaration of Helsinki and was approved by the Ethics Committee of Shiraz University of Medical Sciences, Iran (IR.SUMS.MED.REC.1398.169). Standard informed consent was obtained from all patients, who gave necessary explanations regarding the study objectives). The present study was conducted on patients over 55 diagnosed with type I AMD-CNV, including new cases and cases treated with anti-vascular endothelial growth factor (anti-VEGF). These cases were referred to Poostachi Ophthalmology Clinic in Shiraz, Iran. The center is an excellent referral in south Iran; this study was conducted between September 2017 and September 2018).

A total of 120 patients (137 eyes) were evaluated. All patients were recalled for examination and imaging. A complete ophthalmological examination, including best-corrected visual acuity (BCVA), slit lamp examination, intraocular pressure, fundus examination, and EDI-OCT, were performed for all patients. The diagnosis was based on a series of funduscopic findings and EDI-OCT (made by the company: Heidelberg. Engineering, Heidelberg, Germany).

### Imaging acquisition

EDI-OCT images were obtained using a Heidelberg-spectralis device (Heidelberg Engineering, Heidelberg, Germany); the device was placed as close to the eye as possible to capture the inverted choroidal image in the choroidal mode setting. This inverted image is displayed on the monitor to match the preset pictures. Transverse and vertical EDI-OCT crosshair scans (6 mm×6 mm) were taken from the center of the fovea. Subfoveal choroidal thickness (SFCT) from the Bruch’s layer to the innermost border of the sclera was measured in both vertical and horizontal axes using an electronic caliper, and their mean value was considered as the choroidal thickness (ChT). Two independent operators performed each measurement so that a mean difference of more than 15% in both eyes was reviewed and matched. The Bruch’s hyperreflective layer is often indistinguishable from RPE in healthy eyes and most eyes with early stages of AMD. Therefore, in these cases, the choroidoscleral interface (CSI) was measured from the outermost hyperreflective limit of the RPE layer to the innermost hyperreflective limit. In eyes with detached RPE, the choroidoscleral and Bruch’s layer interface was measured, and the detached portion was not counted in the measurement. As choroidal structures exhibit diurnal variations, all EDI-OCT scans were performed between 9:00 AM and 12:00 MD. Images with 1*1 pixels were used for qualitative analysis, and the SFCT was calculated in 1*1 microns.

### Exclusion criteria

Exclusion criteria were (1) Severe ocular media opacity, (2) Spherical equivalent greater than ± 6.00, (3) Immeasurable choroidal thickness due to poor identification of the chorioscleral interface, (4) The presence of other retinal vascular disorders (e.g., macroaneurysm, proliferative diabetic retinopathy, central retinal vascular occlusion), (5) Eyes with any evidence of type 2 or type 3 neovascularization, (6) Myopic choroidal neovascularization, (7) Other macular disorders such as angioid streaks and retinal detachment, (8) History of previous ocular surgery and intervention other than uncomplicated cataract surgery were excluded from the study).

Also eyes with findings suspicious for polypoidal choroidal vasculopathy (PCV) based on prior studies, such as; any presence of an orange nodule or extensive subretinal hemorrhage on fundus photography, sharp-peaked PED or sub-RPE ring-like lesion on cross-sectional OCT, as well as complex RPE elevation on En face OCT were also excluded from the study [14].

### Inclusion criteria

All patients with a diagnosis of type I (AMD-CNV) were divided into two different groups according to the below criteria: Patients were enrolled in the pachychoroid neovasculopathy (PNV) group if all of the following criteria were met [8, 9, 12, 25]:

1. Both eyes have a subfoveal choroidal thickness (SFCT) of 300 μm or more.
2. Absence of drusens or drusen-like deposits (corresponding to Age-Related Eye Disease Study) in both eyes or presence of pachydrusen, which presented as a singular lesion, more significant than 125 μm, with a scattered outer border.
3. Presence of central serous chorioretinopathy (CSCR), or pachychoroid pigment epitheliopathy (PPE) (included RPE abnormality regardless of CNV lesion, the presence of dilated choroidal vessels, or choroidal thickening below the type 1 CNV, or thinning of the choriocapillaris can be noted over the pachyvessel area or a history of CSCR) in each eye.

Patients were enrolled in the Neovascular AMD group if the following criteria were encountered:

1. Patients with CNV and other findings corresponding to Age-Related Eye Disease Study levels 2, 3, and 4 (extensive hard drusen, soft drusen [intermediate: between 63 and 125 μm and large > 125 μm] [1, 21], focal hyperpigmentation, or geographic atrophy).
2. subfield choroidal thickness (SFCT) < 300 μm in either eye.
3. Absence of CSC or PPE characteristics in recorded medical history and fluorescein 166 angiography or OCT images.

### Statistical methods

Statistical analysis was performed with SPSS software (IBM Corp. Released 2017. IBM SPSS Statistics for Windows, Version 25.0. Armonk, NY: IBM Corp). Descriptive statistics were first calculated for all variables of interest. Mean, and standard deviation (SD) values were calculated for continuous variables, while frequency and percentage were calculated for categorical variables. At first, the normality of the variables was checked. For comparison of the groups, independent T-test and Pearson correlation tests were used for continuous variables and Chi-square tests for categorical data. A P-value of less than 0.05 was regarded as statistically significant.

## Results

Overall, the present retrospective cross-sectional study was carried out on 120 patients (137 eyes) diagnosed with type I AMD-CNV archived in Poostchi ophthalmology clinic, Shiraz, Iran. After complete examinations, including re-examination and imaging findings, 94 patients (106 eyes) were assigned to the AMD group, and 26 patients (31 eyes) were to the PNV group. Hence, 20% of patients with AMD-CNV undergoing treatment had PNV, which was mistakenly categorized as AMD.

In AMD and PNV groups, the mean age was 74.3 ± 8.2 and 63 ± 7.7 years old. The minimum and maximum ages of patients in AMD and PNV groups were 60–94 and 58–90 years, respectively. There was a significant correlation between age and AMD-CNV occurrence based on univariate analysis (P < 0.001).

Regarding sex, 53.2% and 57.7% of AMD and PNV patients were male, respectively, but there was no significant difference between the two groups (P = 0.54). Moreover, 19.2% and 12.8% of patients with AMD and PNV had bilateral involvement, respectively, which was not significantly different (P value = 0.08).

**Fig. 1:**
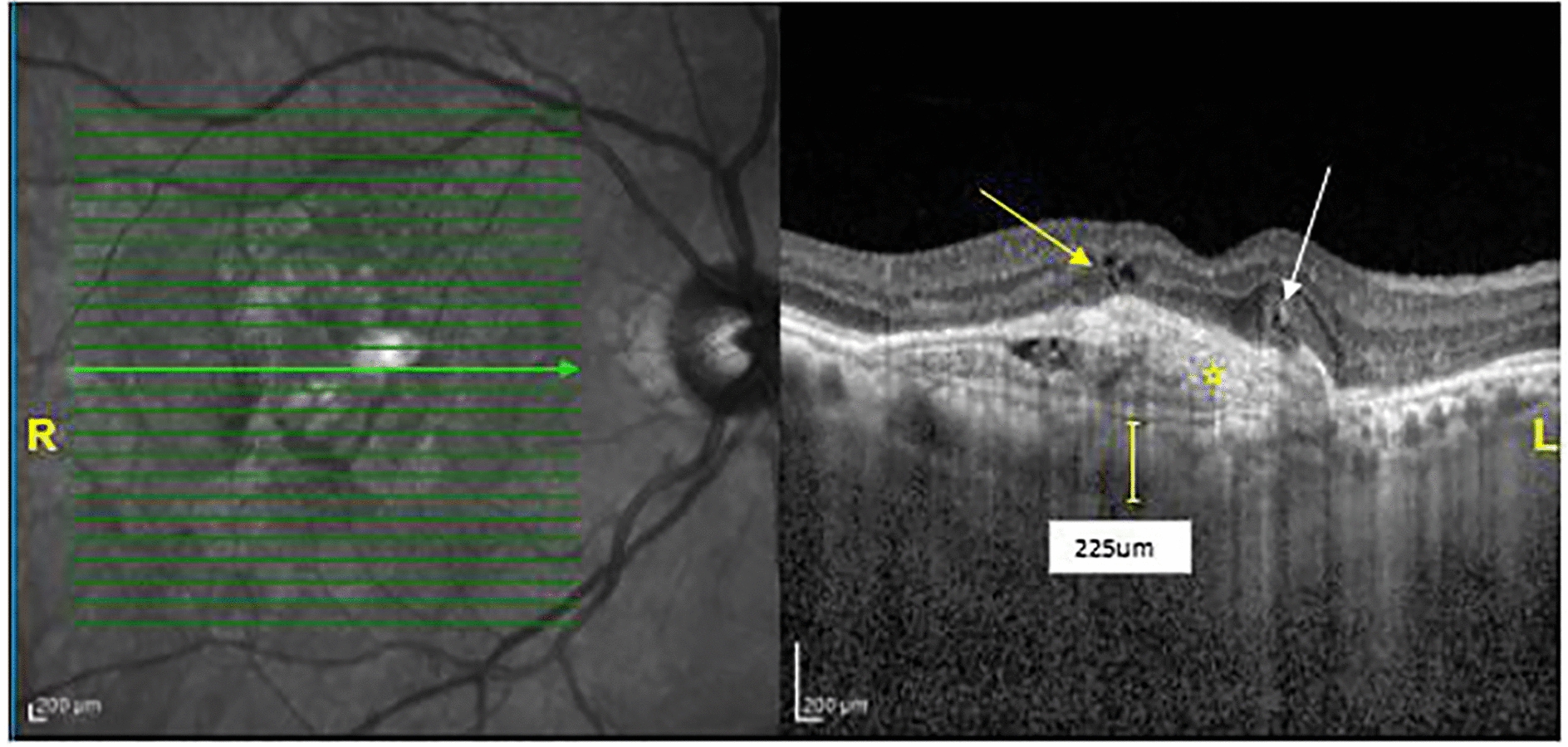
Macular EDI-OCT of a patient with wet AMD. Infrared Reflectance (IR) image (R) and EDI-OCT B‑scan image at the level of the green arrow in the foveal area (L). A small amount of intraretinal fluid (yellow arrow), outer retinal tubulation (white arrow), and subretinal hyperreflective material (asterisk) due to choroidal neovascularization overlying a thin choroid are shown

**Fig. 2:**
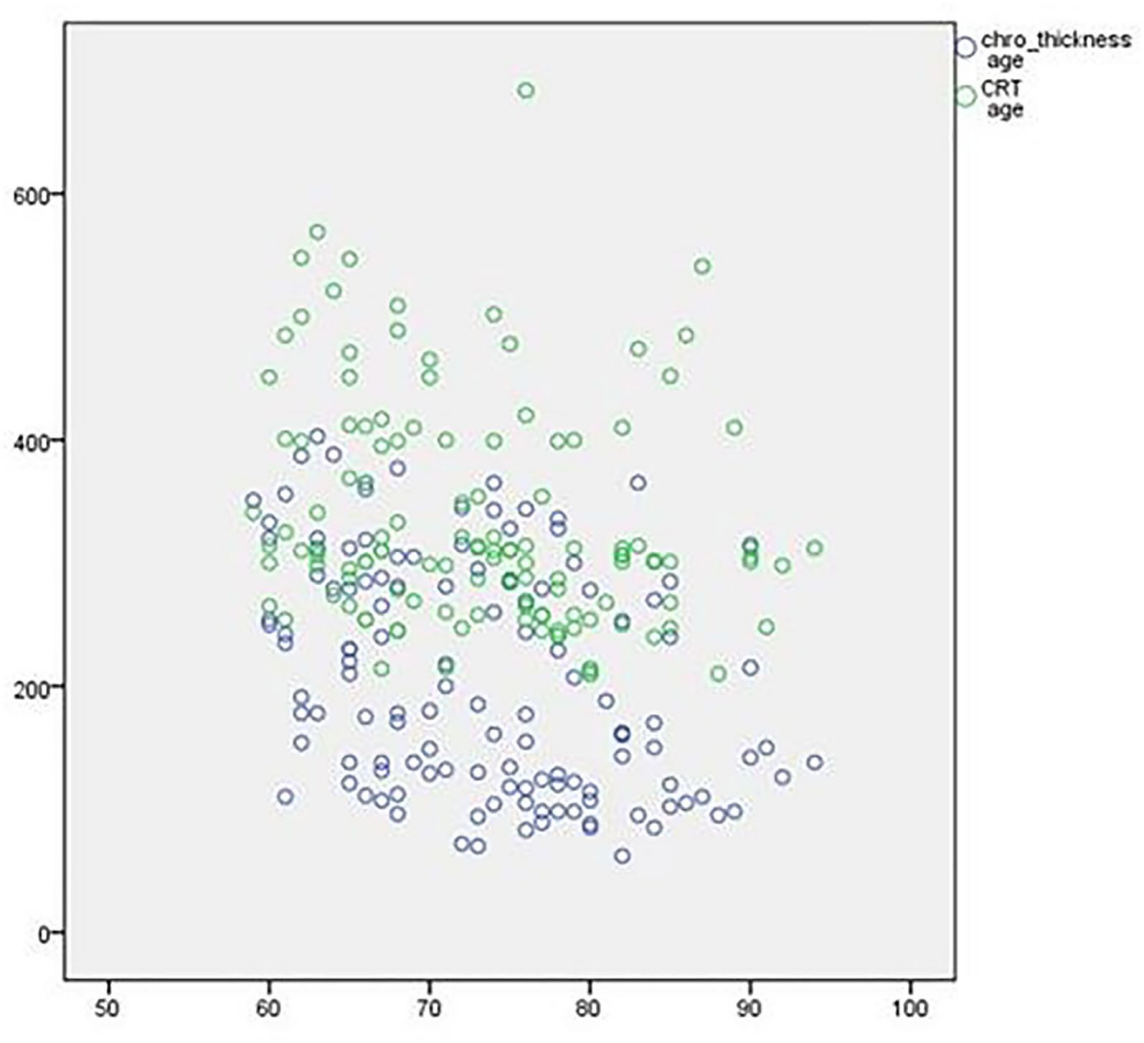
This graph [vertical axis thickness (µm) and the horizontal axis age(year)] shows the distribution of patients according to sub-field choroidal thickness and central retinal thickness (CRT) regarding to patients ages

The mean SFCT in the PNV group was 342 ± 39 μm, which was significantly higher than AMD group (173.8 ± 93 μm) (P < 0.001). Also, the minimum and maximum SFCT in AMD and PNV patients were 62–300 μm and 305–403 μm, respectively. In other words, SFCT was more than 300 μm in all patients with PNV, which was the leading indicator for diagnosing pachychoroid vasculopathy. The macular B-scan OCT of a patient with wet AMD is shown in Fig. 1. Also, in contrast to the aging pattern, the Pearson correlation showed by aging, SFCT dropped dramatically (P-value 0.000, r = 0.36) (Fig. 2)

The mean central retinal thickness (CRT) in the AMD and PNV groups was 324.3 ± 86 μm and 358.8 ± 102 μm, respectively, which was not statistically significant (P = 0.12).

Subretinal fluid (SRF) was higher in eyes with PNV (38.5%) than in eyes with AMD (30.9%). Nevertheless, this difference was not statistically significant (P = 0.84). In contrast, drusen were found in 69.9%, and pachydrusen was seen in 24% of the cases with AMD and PNV, respectively, which was statistically significant (P = 0.01). Also, subfoveal scarring was observed in 65.6% and 28% of AMD and PNV patients, respectively, which was statistically significant (P = 0.03).

The subfoveal atrophy was not significantly different between the two groups (P value = 0.65).

**Fig. 3:**
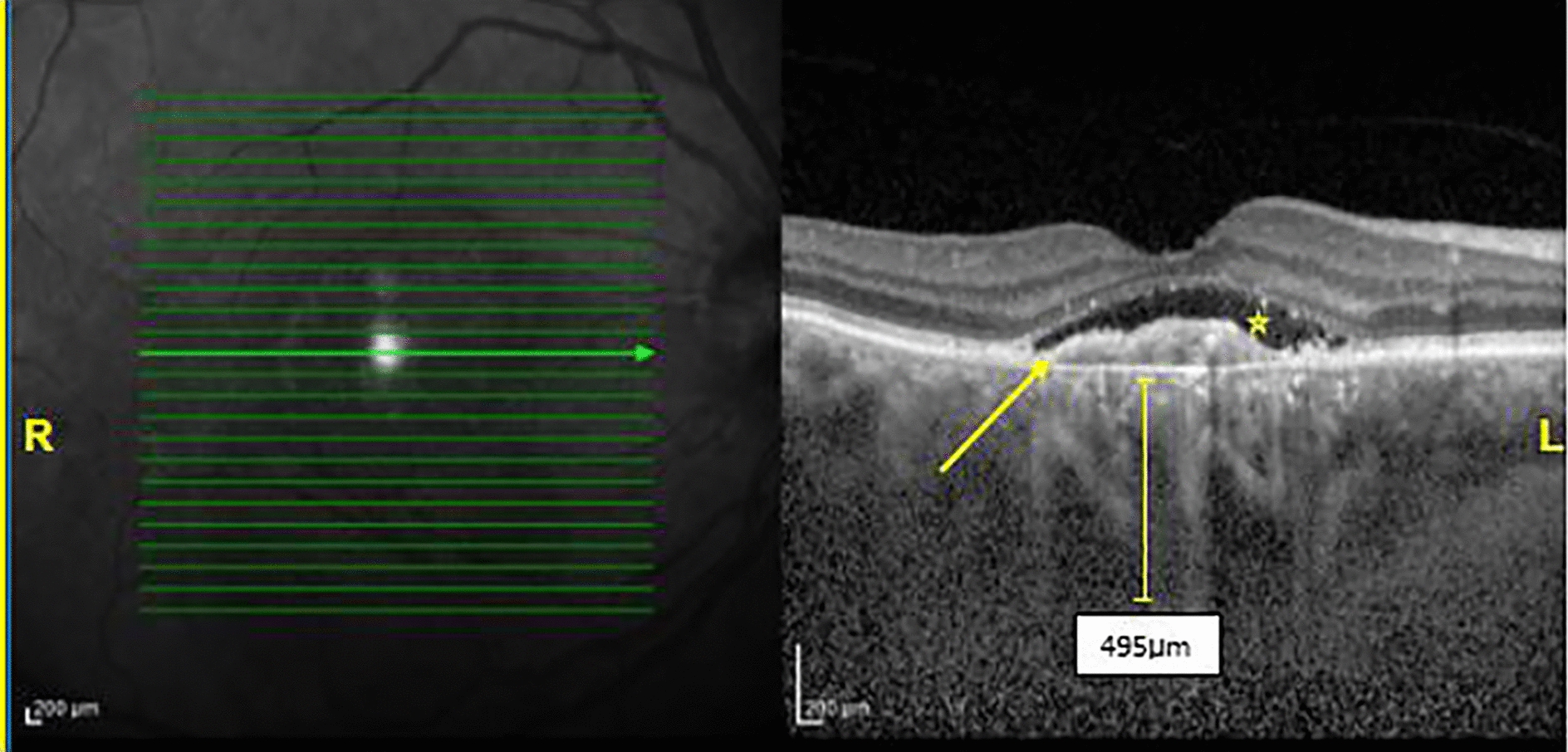
EDI-OCT of a patient with the diagnosis of pachychoroid neovasculopathy (PNV). **R**. The IR image of right eye, the green arrow shows the level of the B-scan image. **L**. EDI**-**OCT B‑scan image demonstrates subretinal fluid (asterisk) and shallow irregular PED (arrow) with a CMT of 320 μm and choroidal thickness of 495 μm

**Fig. 4:**
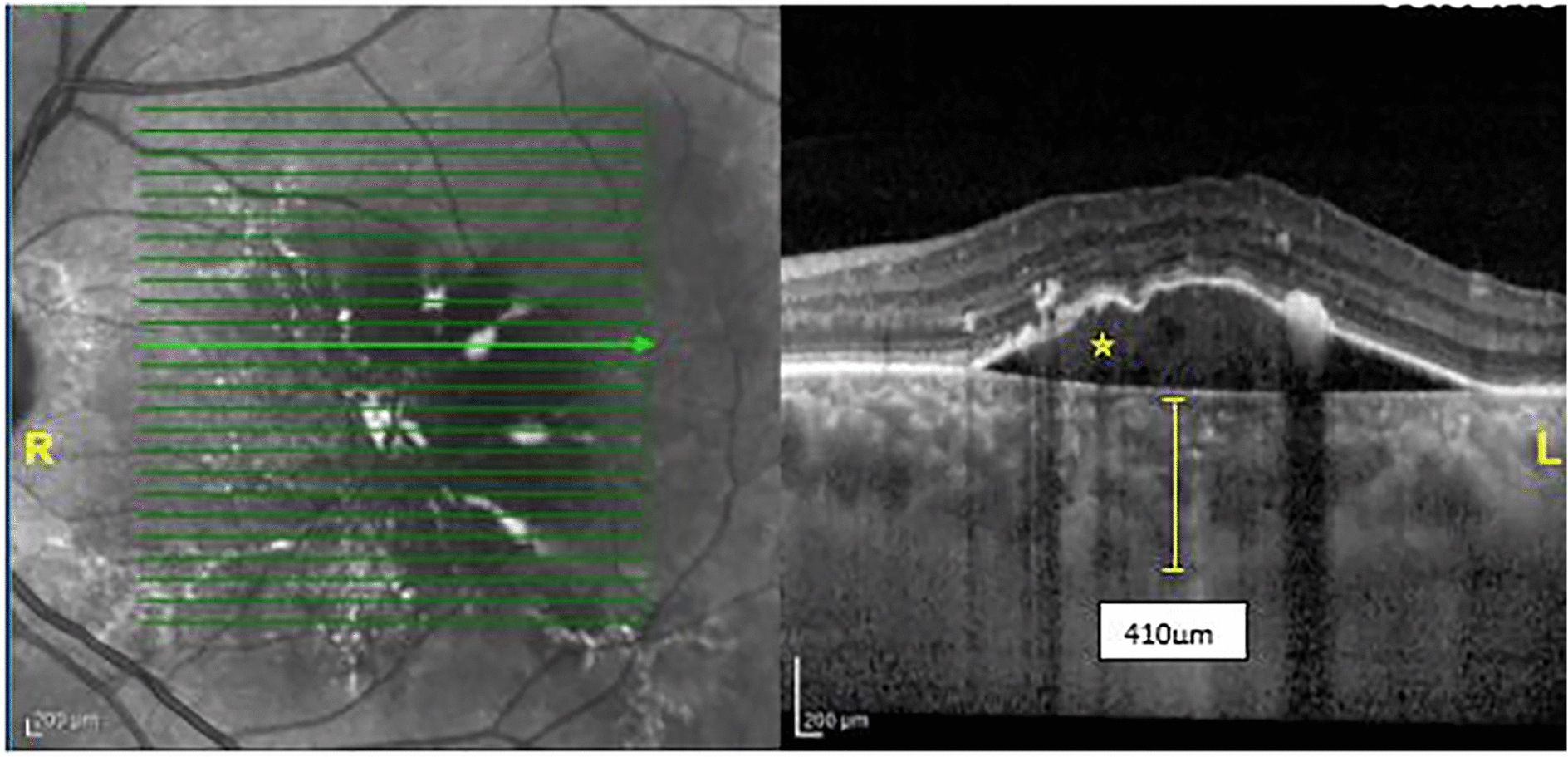
Macular EDI-OCT of a patient with PNV. Infrared Reflectance (IR) image (**R**) and EDI-OCT B‑scan image at the level of the green arrow in the foveal area (**L**). vascularized serous PED (asterisk) is present with CMT of 452 μm and choroidal thickness of 410 μm

Subretinal hyper-reflective material (SHRM) was also seen in 54.8% and 44% of patients in the AMD and PNV groups, respectively, which was not statistically significant (P = 0.33). Figures 3 and 4 demonstrate OCT findings of patients with PNV.

Summarized OCT findings of patients in two groups of PNV and wet AMD are compared in Table 1.

**Table 1:** Comparing OCT findings of patients in two groups of PNV and wet AMD.

OCT findings	PNV group	Wet AMD group	P value
Choroidal thickness	342 ± 27 µ	173.89 ± 69 µ	0.001^#^
Central retinal thickness	358.85 ± 102 µ	324.34 ± 86 µ	0.3
Subretinal fluid	38.5%	30.9%	0.4
Kinds of Drusen **	69.9%	24%	0.000^#^
Subfoveal scarring	28%	65.6%	0.001^#^
Subfoveal atrophy	44%	58.1%	0.2
Subretinal hyper-reflective material	44%	54.8%	0.3
Subretinal hemorrhage*	40%	26.9%	0.2

*PNV*:pachy choroid neovasculopathy,*AMD *aged related macular degeneration

*confirmed with clinical examination,

** detail was described in text(drusen in AMD versus pachydrusen in PNV),

^#^statistically significant

Considering the two cumulative indices of average SFCT > 250 μm and the absence of drusen, PNV would be differentiated from AMD patients in more than 86% of cases. If SFCT is assumed to be 300 μm, this value will reach 100%. If we ignored SFCT and considered the cumulative indices of the absence of drusen and subfoveal scarring, the above percentage was estimated to be about 70%. Moreover, the average number of intravitreal injections of anti-VEGF required in the AMD and PNV groups was estimated to be about 5 ± 1.7 and 3 ± 1.1, respectively, which was statistically significant (P < 0.02).

## Discussion

Based on different studies, the prevalence of PNV is estimated at 22.3–61.6% among the Asian population and 8–13% among the Caucasian population [15–17, 38, 39]. Regarding AMD as the third leading cause of blindness in East Asia and increasing the number of AMD cases with the population aging, its early and late stages prevalences are estimated to be 1.4–37.9% and 0.1–7.3%, respectively [18]. In various studies, especially older studies, PNV was more commonly referred to as the exudative subtype of AMD. Still, with advances in multimodal imaging and genetic studies, this disease was classified as part of the pachychoroid disease spectrum [17, 19, 38]. PNV may be more prevalent than previously assumed, so distinguishing it from AMD-CNV is essential. Although there is still no definite consensus on the PNV classification, most related studies emphasize the need to differentiate PNV from wet AMD [17–19]. According to these studies, in the Asian population, unlike Western people, PNV is more frequently misdiagnosed as AMD-CNV [15, 17, 19]. Miyake et al. reported that within 200 consecutive Japanese patients with neovascular AMD, only 80.5% were affected by neovascular AMD; the other 19.5% were instead influenced by PNV [17]. In the present study, one-fifth of our patients with PNV was misdiagnosed as AMD-CNV. Since the initial diagnosis and final preoperative examination are performed solely by retinal specialists in our ophthalmology center and considering Shiraz as the referral ophthalmology and retinal disease center in the south of Iran, this rate of misdiagnosis is significant and should be considered. The importance of this finding is debatable from several points of view. Firstly, CNV as a problem requiring treatment is seen in both diseases, but the pathogenesis of these diseases is entirely different. Therefore, the course of treatment, prognosis of patients, care process, and follow-up of the contralateral eye are other in the two diseases [17]. In addition, unlike AMD-CNV, photodynamic therapy (PDT) plays a vital role in treating PNV as a part of pachychoroid spectrum diseases. Hence, introducing appropriate diagnostic factors with higher sensitivity and specificity to differentiate PNV from AMD-CNV is crucially important.

According to the present study’s findings, the mean age of people with AMD was higher than PNV, with a significant difference between the two groups. It is important to note that, as mentioned by several studies, PNV patients are younger than that of AMD- CNV patients, so the prevalence of AMD is 10% in the population below 65 years old and 25% in the population aged 75 and over in developed countries [21, 22]. It should be considered that some of the patients diagnosed with PNV were even over 85 years old in the present study, so old age does not rule out the diagnosis of this disease. Nevertheless, based on various studies, it can be said that the younger generation mainly contributes to PNV diagnosis instead of AMD diagnosis [17, 19, 22].

According to various studies [20–22], one of the most important demographic and clinical factors differentiating PNV from AMD-CNV were the lower prevalence of bilateral involvement (5.9 vs. 24.1% respectively) and greater participation among men (78.5% vs. 63%, respectively) in the PNV. Consistent with previous findings, in our study, the prevalence of bilaterality in the PNV group was lower than in the AMD-CNV group (12.8% vs. 19.2%). However, this difference was not statistically significant. Regarding sex, men are more susceptible to AMD, according to some studies [23, 25], and in some studies [19, 26], female sex has been suggested as a protective factor for both PNV and AMD. However, there was no significant difference between the two groups regarding sex in the present study.

In comparisons of anatomical differences between PNV and AMD in epidemiological studies, increased choroidal thickness appears to be a prevalent factor. [38, 39] Regarding diagnosing pachychoroid spectrum disorders, the subfoveal choroidal thickness reported in the literature ranges from 200 to 395 μm [3, 6, 19]. To make the criteria mentioned above more inclusive, we chose the higher thickness value (more than 300 μm) in this study; we considered the subfoveal choroidal thickness (SFCT) measures as the most critical factor differentiating PNV from AMD-CNV. However, it should be noted that this index loses its reliability in the end stages of this disease following choroidal atrophy [22, 23].

In some older studies, ICGA is considered the gold standard imaging modality for diagnosing pacychoroid spectrum diseases and PCV [27, 28]. ICGA can demonstrate choroidal hyperpermeability, pachyvessels, choroidal filling defects, delayed arterial filling, and patchy hyperfluorescent areas that often correspond to leakage sites on FA [29].

However, ICGA is an invasive, burdensome, and time-consuming procedure with limited availability. It also carries the risk of systemic adverse reactions [30] So, ICGA is usually not performed routinely in most ophthalmic practices. Moreover, it is crucial to note that eyes with choroidal hyperpermeability on ICGA usually have increased SFCT. But not all looks with thick choroids reveal choroidal hyperpermeability on ICGA [29]. Altinisic et al. and arf et al. [36, 37] compared OCTA features of PNV with AMD-CNV in separate studies. They reported that neither morphology nor area and vessel density detected by OCTA differed from PNV to AMD-CNV.

Despite some limitations, we propose EDI-OCT as an accurate, non-invasive, and accessible imaging modality for distinguishing PNV from typical AMD-CNV.

In the present study, indices such as the presence of subfoveal atrophy and subretinal hemorrhage (SRH) were assessed in both groups. SRH was seen in 40% of patients with PNV compared to 26.9% in AMD ones. Our findings mimicked those of the Tagawa et al. study. They reported 20.2% SRH occurrence in their study population. It also mentioned that SRH was seen more frequently in the polypoidal lesion-positive group than in the polypoidal lesion-negative group. We have had more significant numbers of patients with SRH because SRH is thought to be positive in any size in our study, whereas Tagawa et al. only pointed out more than 4-disc areas in SRH as positive cases [33].

The prevalence of subretinal scarring in our AMD-CNV patients was twice as high as in PNV patients, according to various studies [19, 22, 26]. This index is one of the essential factors differentiating these two diseases. It is one of the leading causes of decreased vision and poor prognosis among AMD-CNV patients compared to PCV-PNV patients. This index was first proposed by Yannuzzi et al. and later confirmed by different studies [24].

Pachydrusen was recently defined by Spaide [34] as a new form of drusen, typically larger than 125 μm. Often had an irregular outer contour distributed over the posterior pole lonely or in the group. That was in only about one-fifth (24%) of PNV patients in the present study, in contrast, typical drusen were found in more than 65% of AMD patients.

Moreover, this characteristic, if associated with a higher SFCT, increased the differentiation of the two diseases by more than 80%. Overall, the results of the present study reveal that the presence of drusen solely does not reject PNV diagnosis. It is essential to distinguish the drusenoid lesion type. It should be noted that the company of reticular pseudo-drusen (RPD) has been reported in both diseases [23, 25]. In one study, the prevalence of RPD in AMD and PNV groups was said to be 9.2% and 2.2%, respectively, where the presence of RPD has been associated with the progression of neovascularization in PNV as well as the progression of geographic atrophy in AMD [23, 25].

Although different pathogenesis for PNV has been speculated, such as choroidal vessel expansion, VEGFs play a different role in PNV compared to neovascular AMD [8, 9]. The mainstay of treatment is based on intravitreal anti-VEGF injections, the same as for neovascular AMD, with a loading dose of three-monthly injections, followed by additional injections if needed. Usually, PNV patients have a more extended retreatment-free period than AMD patients and require fewer injections during the follow-up [17, 35]. In our study, the treatment was the same (intravitreal bevacizumab 1.25 mg) for all patients. Still, the average number of injections required in PNV patients was almost half of AMD-CNV patients (3 vs. 5) during patient follow-ups. According to most studies [31, 32], consistent with the present study, this number is lower in PNV patients than in AMD-CNV patients. Also, PNV patients did not need to be injected for a more extended period [22, 23].

On the other hand, various studies have confirmed the adjuvant role of PDT in pachychoroid disorders, unlike AMD-CNV, especially in patients who do not respond well to anti-VEGF [20–23]. There are limitations in our study that should be mentioned. Relatively small sample sizes for patients and the study’s retrospective design might restrict the statistical power for detecting the differences in the factors influencing the outcomes. Also, given the cross-sectional design of the current study, we could not assess the treatment response and visual effect of the two study groups. Furthermore, we did not perform ICGA for our study subjects due to the unavailability of an indocyanine green vial in our country during the study period.

## Conclusion

This study revealed that about one-fifth of wet AMD patients were actually pachychoroid neovasculopathy and often were misdiagnosed. These patients were younger and developed less subretinal scarring. Thus, the disorder must be considered an important differential diagnosis of AMD-CNV. Moreover, the prognosis is better and the retreatment-free period of the patients is longer in PNV. So, recognizing and identifying the characteristics of the pachychoroid neovasculopathy could potentially avoid mismanagement or over-investigation.

## Data Availability

Data is available upon request from author.

## Abbreviations

AMD: Age-related macular degeneration
PNV: Pachychoroid neovasculopathy
CSI: Choroidoscleral interface
ICG: Indocyanine Green Angiography
CSCR: Central serous chorioretinopathy
PPE: Pachychoroid pigment epitheliopathy
BCVA: Best-corrected visual acuity
OCT: Optical coherence tomography
VEGF: Vascular endothelial growth factor
